# Defining Critical View of Safety During Laparoscopic Cholecystectomy: The Preoperative Predictors of Failure

**DOI:** 10.7759/cureus.37464

**Published:** 2023-04-12

**Authors:** Rahul Gupta, Archana Khanduri, Arvind Singh, Harshdeep Tyagi, Rahul Varshney, Nagendra Rawal, Ujjwal Daspal, Sudhir K Singh, Parikshit Morey, Pradip Pokharia

**Affiliations:** 1 Gastrointestinal Surgery, Synergy Institute of Medical Sciences, Dehradun, IND; 2 Gastroenterology, Synergy Institute of Medical Sciences, Dehradun, IND; 3 Anaesthesiology, Synergy Institute of Medical Sciences, Dehradun, IND; 4 Anesthesia and Critical Care, Synergy Institute of Medical Sciences, Dehradun, IND; 5 Radiology, Synergy Institute of Medical Sciences, Dehradun, IND

**Keywords:** neutrophil percent, lymphocyte percent, critical view of safety, cholecystectomy, gallstones

## Abstract

Background

Defining critical view of safety (CVS) is one of the most crucial steps during laparoscopic cholecystectomy (LC). This study aimed to determine the preoperative predictors of failure to achieve CVS during LC.

Methods

All patients undergoing LC from December 2020 to July 2022 were prospectively included.

Results

There were 180 females and 93 males. CVS was achieved during LC in 238 (87.2%) patients. Conversion to open surgery was performed for 11 patients. Bile leak occurred in three patients which resolved spontaneously. No patient developed bile duct injury. On univariate analysis, age, male sex, American Society of Anaesthesiologists (ASA) grading, Murphy’s sign, emergency surgery, neutrophil percentage, lymphocyte percentage, gallbladder wall thickness > 3mm, and impacted gallstone on abdominal ultrasound were predictors of failure to achieve CVS. On multivariate analysis, neutrophil and lymphocyte percentages were independent predictors of failure to achieve CVS. Patients in whom CVS could not be achieved had significantly longer operative time, higher blood loss, complications, and hospital stays.

Discussion

Inability to achieve CVS during LC can be predicted preoperatively using various parameters including neutrophil and lymphocyte percentages. Such cases must be operated by senior surgeons or referred to experienced general or hepatobiliary surgeons for cholecystectomy to avoid bile duct injury. The proposed algorithm can help in intraoperative decision-making in difficult cases.

## Introduction

Laparoscopic cholecystectomy (LC) is one of the most commonly performed surgical procedures worldwide. Bile duct injury (BDI) is the most dreadful complication of LC. The best way to prevent BDI during LC is to perform meticulous dissection and display critical view of safety (CVS) as pioneered by Strasberg et al. in 1995 [1]. However, it may not be possible to display CVS in certain cases due to abnormal anatomy, adhesions, inflammation, stone impaction, etc. In such cases, the risk of BDI increases. Hence, such cases should be operated by expert general or hepatobiliary surgeons at high-volume centers [2]. Several studies have been conducted to determine the predictors of difficult cholecystectomy [3-6]. However, the most frequently used parameters to define surgical difficulty have been operative time, conversion to open, intraoperative difficulty scores, etc [4,5]. These parameters are affected by various surgeon-based and patient-based factors. However, the most important factor affecting intraoperative decision-making and postoperative outcomes is the ability to display CVS. Proceeding with cholecystectomy without achieving CVS is the most frequent cause of BDI. Moreover, most of the studies have combined preoperative parameters with intraoperative findings to predict operative difficulty. We conducted this study to determine preoperative predictors of difficult Calot’s anatomy leading to failure to achieve CVS during LC so that patients having such risk factors can be referred to expert general or hepatobiliary surgeons. We have also studied the impact of failure to achieve CVS on the outcomes of LC and discussed the alternative techniques that can be employed to perform cholecystectomy when CVS cannot be displayed.

## Materials and methods

Patients and objectives

This single-center prospective cohort study was conducted at the Department of Gastrointestinal Surgery, Synergy Institute of Medical Sciences, Dehradun, India. The study protocol was approved by the Ethics Committee of Shri Guru Ram Rai Institute of Medical & Health Sciences, Dehradun (SGRR/IEC/17/21). All patients provided written informed consent for participation in this study. All consecutive patients (age > 12 years) undergoing elective or emergency LC for gallstone disease from December 2020 to July 2022 were included. Patients with LC for other causes, Mirizzi syndrome, upfront open cholecystectomy, and cholecystectomy combined with common bile duct (CBD) exploration were excluded. Acute cholecystitis (AC) was diagnosed preoperatively using Tokyo Guidelines 2018 [7].

The primary objective was to determine the preoperative predictors of failure to achieve CVS. The secondary objectives were to determine the accuracy of intraoperative difficulty scoring systems in predicting failure to achieve CVS and impact of failure to achieve CVS on postoperative outcomes.

The preoperative parameters recorded in this study were age, sex, body mass index (BMI), comorbidities, American Society of Anaesthesiologists (ASA) grading, previous endoscopic retrograde cholangiopancreatography (ERCP), history of acute pancreatitis, and clinical findings such as right upper quadrant tenderness. Blood investigations such as total leucocyte count (TLC), neutrophil percentage, lymphocyte percentage, neutrophil/lymphocyte ratio (NLR), and systemic immune-inflammation index (SII) (neutrophil count x platelet count/lymphocyte count in cells/L) were also recorded. The findings of abdominal ultrasound included in this study were gallbladder (GB) status (contracted vs. distended), GB wall thickness (< 3mm vs. > 3mm), number of stones (single vs. multiple), presence of impacted GB stones, pericholecystic fluid, and maximum stone diameter.

Surgical technique

All patients received prophylactic antibiotics at the time of induction. LC was performed under general anesthesia using the standard four ports. In some cases, three or five ports were used based on intraoperative surgical difficulty. The standard protocol in this study was to clearly define CVS in all cases before dividing the cystic duct and artery. The dissection was performed using a laparoscopic hook (Om Surgicals, Mumbai, India) and a Maryland dissector (Om Surgicals, Mumbai, India) in most cases. In some cases, a harmonic scalpel (Ethicon, Mumbai, India) was used to facilitate dissection. The cystic artery and duct were clipped by Hem-o-lok polymer clips (Dextron Medical Solutions, Surat, India) in most cases. All the surgeries were performed by a single surgical team with the operating surgeon having experience of more than five years in performing LC. The operative time was calculated from the start of port insertion to the skin suturing of port sites. The intraoperative surgical difficulty was determined using the modified Nassar grading [8], Sugrue scoring system [9], and Parkland grading [10]. LC was converted to an open procedure in case of excessive bleeding, inability to define CVS, inability to proceed safely, and bowel injury. Abdominal drain was placed in the subhepatic space in difficult cases.

Postoperative care 

All patients received standard postoperative care including intravenous fluids, antibiotics, and analgesics. The patients were monitored in-hospital and on an outpatient basis after discharge for postoperative complications and the same were recorded. 

Statistical analysis

The patients were divided into two groups for comparison: ‘CVS’ and ‘no CVS’ groups. Continuous variables were expressed as mean + standard deviation and compared using the Student t-test. Categorical variables were expressed as frequency (%) and compared by Chi-square or Fischer test as appropriate. Binary logistic regression analysis was performed to determine the independent predictors of inability to achieve CVS. All statistical analyses were performed using IBM SPSS Statistics for Windows, Version 23 (Released 2015; IBM Corp., Armonk, New York, United States)

## Results

A total of 288 patients underwent cholecystectomy during the study period. Out of these, 15 patients were excluded due to the following reasons: upfront open cholecystectomy (n = 6), LC combined with CBD exploration (n = 5), and Mirizzi syndrome (n = 4). Finally, 180 females and 93 males were included in this study. The mean age of the whole cohort was 46.55 + 15.12 years. The mean BMI was 25.42 + 4.36. One or more comorbidities were present in 63 patients. Emergency surgery was performed in 77 (28.2%) patients. Preoperative ERCP for choledocholithiasis was performed in 41 patients. Acute gallstone pancreatitis was present in 18 patients. AC was suspected preoperatively in 62 patients. On abdominal ultrasound, contracted GB, pericholecystic fluid, stone impaction in the Hartmann’s pouch or cystic duct, single stone, and GB wall thickness > 3mm were noted in 29, 14, 27, 71, and 64 patients, respectively. 

CVS was achieved in 238 (87.2%) patients (elective - 91.3% and emergency - 76.2%). CVS could not be achieved in 35 patients due to dense adhesions (n = 20), stone impaction (n = 7), overhanging liver segment IVb (n = 7), and multiple cystic vessels (n = 1). Out of these 35 patients, laparoscopic total cholecystectomy, open total cholecystectomy, and open subtotal cholecystectomy were performed in 24, 9, and 2 patients, respectively.

On univariate analysis, older age, emergency surgery, higher neutrophil percentage, lower lymphocyte percentage, gallbladder wall thickness > 3mm, and impacted stone on ultrasound were risk factors for inability to achieve CVS (Table 1). 

**Table 1:** Comparison of preoperative parameters between the two groups – univariate and binary logistic regression analysis. ASA: American Society of Anaesthesiologists; ERCP: endoscopic retrograde cholangiopancreatography; RUQ: right upper quadrant

Parameter	CVS group (n = 238)	No CVS group (n = 35)	Univariate analysis (P value)	Multivariate analysis (P value)	Odds ratio (95% CI)
Age (years) (mean +SD)	44 + 15	50 + 13	0.013	0.342	0.984 (0.951-1.017)
Male sex	76 (32)	17 (49)	0.052	0.950	1.028 (0.428-2.469)
Body mass index	25.34 + 4.29	26.12 + 4.98	0.344	-	-
Comorbidities	51 (22)	12 (34)	0.098	-	-
ASA I	112 (48)	11 (32)	0.082	-	-
ASA II	89 (38)	17 (49)	0.205	-	-
ASA III	32 (13)	6 (17)	0.555	-	-
ASA IV	2 (1)	1 (2)	0.285	-	-
Emergency surgery	59 (25)	18 (51)	0.001	0.072	0.416 (0.160-1.081)
Preoperative diagnosis of acute cholecystitis	50 (22)	12 (35)	0.079	-	-
Previous ERCP	36 (15)	5 (15)	0.897	-	-
Previous acute pancreatitis	15 (6)	3 (9)	0.712	-	-
RUQ tenderness	24 (10)	8 (24)	0.023	0.823	1.145 (0.350-3.752)
Total leucocyte count	8106 + 2678	8492 + 2317	0.427		
Neutrophil percent	64 + 10	69 + 9	0.025	0.003	1.035 (1.012-1.059)
Lymphocyte percent	28 + 9	24 + 8	0.005	0.001	1.055 (1.023-1.088)
Neutrophil lymphocyte ratio	2.95 + 2.77	3.98 + 5.17	0.079		
Platelet count	220 + 75	230 + 83	0.471		
Systemic inflammatory index	640.4 + 636.34	774.1 + 801.32	0.272		
Hepatomegaly	51 (24)	10 (32)	0.303		
Contracted GB	26 (11)	3 (9)	1.000		
GB wall thickness >3mm	49 (23)	15 (48)	0.003	0.097	0.462 (0.186-1.150)
Impacted stone	18 (8)	9 (28)	<0.001	0.143	0.447 (0.152-1.313)
Pericholecystic fluid	11 (5)	3 (9)	0.402		
Single stone	63 (28)	8 (24)	0.641		
Maximum stone diameter	12 + 6	14 + 8	0.172		

On binary logistic regression analysis, only neutrophil percent and lymphocyte percent were found to be independent risk factors for failure to obtain CVS (Table 2). 

**Table 2:** Comparison of intraoperative parameters and postoperative outcomes between the two groups.

Parameter	CVS group (n = 238)	No CVS group (n = 35)	P value
Modified Nassar grade			
I	81 (36)	2 (7)	<0.001
II	79 (35)	10 (36)	0.585
III	48 (21)	7 (25)	0.981
IV	17 (8)	9 (32)	<0.001
Sugrue score			
1-2	118 (52)	6 (21)	<0.001
3-4	60 (27)	10 (36)	0.670
5-7	47 (21)	12 (43)	0.051
8-10	0 (0)	0 (0)	-
Parkland grade			
1	83 (37)	2 (7)	<0.001
2	93 (41)	10 (36)	0.231
3	35 (16)	7 (25)	0.417
4	9 (4)	5 (18)	0.008
5	5 (2)	4 (14)	
Operative time (min)	50 + 20	84 + 37	<0.001
Estimated blood loss (ml)	28 + 34	80 + 96	0.003
Conversion to open	0 (0)	11 (31)	<0.001
Duration of hospital stay (d)	1.87 + 1.4	2.94 + 2.17	0.007
Overall complications	22 (9)	7 (20)	0.054
Mortality	1	0	
Bile leak	1	2	
Reoperation	1	0	
Wound infection	2	1	
Urinary retention	7	2	
Hospital acquired pneumonia	1	1	
Gastroparesis	1	0	
Port site bleeding	1	0	
Port site hematoma	2	1	
Atrial fibrillation	1	0	

**Figure 1:**
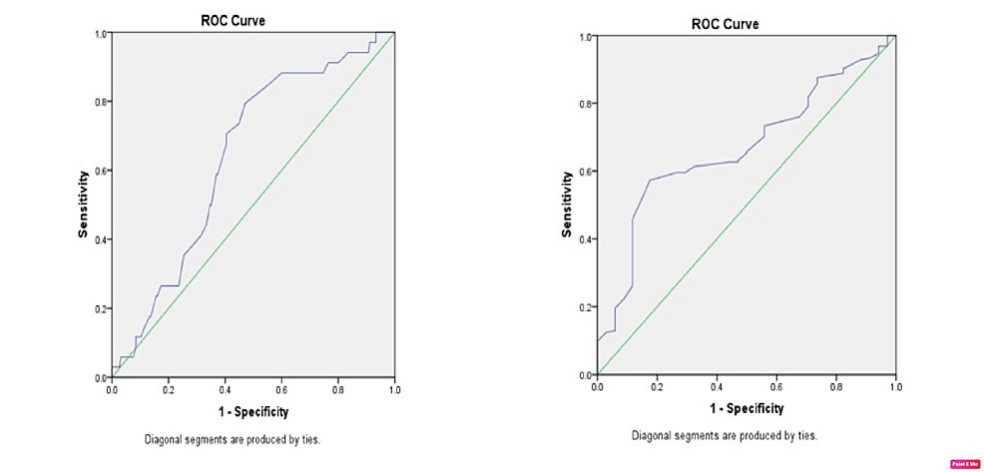
(A) Receiver operating curve (ROC) analysis for neutrophil percent. The area under the curve was 0.641 (95%CI: 0.552 – 0.729). (B) Receiver operating curve analysis for lymphocyte percent. The area under the curve was 0.668 (95%CI: 0.581 – 0.755).

On ROC analysis, neutrophil percentage >65% had sensitivity of 70.6% and specificity of 60% while lymphocyte percentage <27% had sensitivity of 62.7% and specificity of 56% in predicting failure to achieve CVS with the area under the curve of 64% and 66.8%, respectively (Figure 1).

CVS could not be achieved in 35 patients. Out of these patients, total LC was completed in 24 patients using the infundibular or fundus first technique. In the remaining 11 patients, open total cholecystectomy (n = 9) was performed after delineating the cystic duct by the fundus first approach and open subtotal cholecystectomy (n = 2) as the cystic duct could not be defined. On comparison of intraoperative findings, all three difficulty scoring systems could predict failure to obtain CVS (Table 2). The mean operative time and estimated blood loss were 63 minutes and 36 ml. The operative time and intraoperative blood loss were significantly higher in the no-CVS group (Table 2). 

The most frequent postoperative complication in this study was urinary retention (11/26) which was treated by catheterization. Three patients developed postoperative bile leak which resolved with conservative treatment (n = 2) and endoscopic biliary stenting (n = 1). Reoperation was required in one patient due to postoperative bleeding from the gallbladder bed. No patient developed major bile duct injury in this study. One patient with multi-drug-resistant gallbladder empyema and cholangitis died in the postoperative period due to sepsis. The mean postoperative hospital stay and complications were significantly lower in the CVS group (Table 2).

## Discussion

The most critical step of LC is to achieve CVS. The difficulty in obtaining CVS is most often determined by the intraoperative findings. In this study, we attempted to determine the difficulty in obtaining CVS using preoperative parameters so that the treating surgeon can decide beforehand whether to operate or refer the patient to a hepatobiliary surgeon. Preoperative determination of difficult CVS can also help the surgeon in counseling the patients regarding higher chances for conversion to open procedure and subtotal cholecystectomy. 

In this study, CVS could not be achieved in about 13% (35/273) of patients. This is similar to that reported by previous studies [11,12]. One of the largest studies conducted in the United Kingdom involving 12,844 patients found age > 40 years, male gender, presence of cholecystitis, choledocholithiasis, thickened gallbladder, preoperative ERCP, and emergency surgery to be independent predictors of difficult LC [3]. A previous study by Khan et al involving 5136 patients reported that patients with stones impacted in Hartmann’s pouch had higher incidence of AC, gallbladder empyema, and Mirizzi syndrome, higher grades of operative difficulty but similar postoperative complications [13]. A study by Jalil et al. also found stone impaction to be the single most important ultrasound finding to predict difficulty of LC [14]. A French study involving 420 patients found male gender, history of AC, absolute neutrophil count, serum fibrinogen, and alkaline phosphatase levels to be predictive of difficult LC [15]. A Japanese study of 584 patients by Shimoda et al. reported that age, serum albumin, neutrophil percent, lymphocyte percent, platelet count, NLR, and history of AC were associated with bailout surgery for cholelithiasis [16]. However, only age, history of AC, and serum albumin levels were significant risk factors in multivariate analysis. In the current study, the preoperative variables associated with failure to achieve CVS on univariate analysis were older age, emergency surgery, higher neutrophil percentage, lower lymphocyte percentage, gallbladder wall thickness > 3mm, and impacted stone on ultrasound as reported by previous studies. However, only neutrophil and lymphocyte counts were found to be independent predictors of failure to achieve CVS on multivariate analysis. The differences in the predictors of difficult LC in various studies are probably due to heterogeneity in the definition of difficult LC and different study populations.

**Figure 2:**
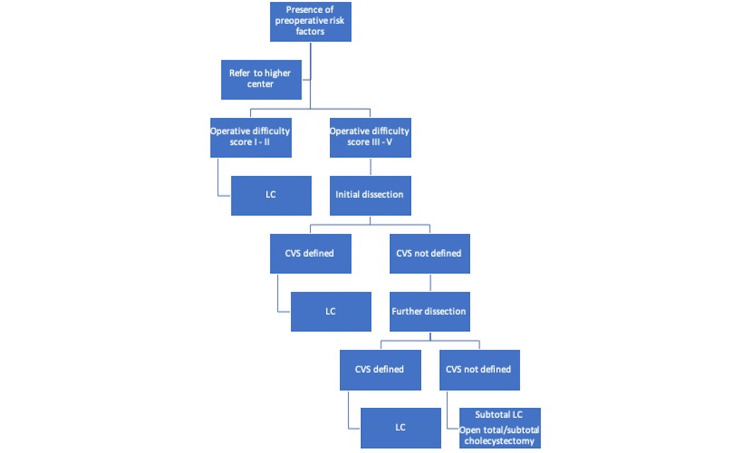
A proposed algorithm to facilitate intraoperative decision-making during laparoscopic cholecystectomy. Operative difficulty is scored using Modified Nassar scale. Initial dissection – separation of omental adhesions and duodenum from gallbladder, gallbladder decompression, opening of anterior and posterior peritoneal folds, removal of cystic node, use of gauze piece for blunt dissection, opening of gallbladder, and removal of impacted gallstones. Further dissection – Fundus first approach, infundibular approach, subserosal dissection, intraoperative conventional or indocyanine green fluorescent cholangiography (if available). CVS: Critical view of safety; LC: laparoscopic cholecystectomy

Among 35 patients in whom CVS could not be achieved, LC was completed successfully in 24 patients by using the infundibular or fundus first technique. Both these techniques have been reported to be safe in patients with difficult LC [17,18]. Other measures recommended by multiple surgical societies for better identification of the biliary anatomy include the use of intraoperative conventional and indocyanine green fluorescent cholangiography [2,11]. However, due to the lack of expertise and equipment, these techniques were not used at our center. Recently, laparoscopic subtotal cholecystectomy has been widely adopted as the preferred salvage technique in difficult cases [19,20]. However, it is associated with significant risk of postoperative bile leak and residual stones in remnant GB [21]. At our center, we preferred to convert to an open procedure if safe LC was not feasible using all possible techniques instead of doing laparoscopic subtotal cholecystectomy. Based on our experience and the existing literature, we propose an algorithm to help surgeons in intraoperative decision-making in difficult cases (Figure 2).

All three scoring systems used in this study to grade the operative difficulty based on the intraoperative findings at the beginning of LC were accurate in predicting failure to achieve CVS as shown in Table 2. The higher the grade, the higher was the risk of failure to achieve CVS. Failure to achieve CVS was associated with higher blood loss, longer operative time, increased risk of conversion to open procedure, higher postoperative complications, and longer hospital stay (Table 2). Similar findings were reported by previous studies [11].

There are some limitations of this study. First, it was a single-center study with a limited sample size. Second, the serum C-reactive protein level was not tested in this study as it is not routinely used in our clinical practice. 

## Conclusions

Ability to achieve CVS is one of the important steps during LC. However, it may not be possible to achieve CVS during LC in every case. Such patients can be identified preoperatively using neutrophil percent, lymphocyte percent, and abdominal ultrasound. These tests are routinely performed in patients before LC and do not involve any extra cost. These patients should be operated by experienced senior surgeons as it may require intraoperative biliary imaging, alternate surgical approaches, or bailout procedures. Preoperative determination of difficult LC can also help to refer such cases to higher centers with available expertise.

## References

[1] Strasberg SM, Hertl M, Soper NJ (1995). An analysis of the problem of biliary injury during laparoscopic cholecystectomy. J Am Coll Surg.

[2] Brunt LM, Deziel DJ, Telem DA (2020). Safe Cholecystectomy Multi-society Practice Guideline and State of the Art Consensus Conference on prevention of bile duct injury during cholecystectomy. Ann Surg.

[3] Nassar AH, Hodson J, Ng HJ, Vohra RS, Katbeh T, Zino S, Griffiths EA (2020). Predicting the difficult laparoscopic cholecystectomy: development and validation of a pre-operative risk score using an objective operative difficulty grading system. Surg Endosc.

[4] Bhandari TR, Khan SA, Jha JL (2021). Prediction of difficult laparoscopic cholecystectomy: an observational study. Ann Med Surg (Lond).

[5] Lee R, Ha H, Han YS, Jung MK, Chun JM (2017). Predictive factors for long operative duration in patients undergoing laparoscopic cholecystectomy after endoscopic retrograde cholangiography for combined choledochocystolithiasis. Surg Laparosc Endosc Percutan Tech.

[6] Bharamgoudar R, Sonsale A, Hodson J, Griffiths E (2018). The development and validation of a scoring tool to predict the operative duration of elective laparoscopic cholecystectomy. Surg Endosc.

[7] Yokoe M, Hata J, Takada T (2018). Tokyo Guidelines 2018: diagnostic criteria and severity grading of acute cholecystitis (with videos). J Hepatobiliary Pancreat Sci.

[8] Nassar AHM, Ashkar KA, Mohamed AY, Hafiz AA (1995). Is laparoscopic cholecystectomy possible without video technology?. https://www.tandfonline.com/doi/abs/10.3109/13645709509152757.

[9] Sugrue M, Sahebally SM, Ansaloni L, Zielinski MD (2015). Grading operative findings at laparoscopic cholecystectomy- a new scoring system. World J Emerg Surg.

[10] Madni TD, Leshikar DE, Minshall CT (2018). The Parkland grading scale for cholecystitis. Am J Surg.

[11] Nassar AH, Ng HJ, Wysocki AP, Khan KS, Gil IC (2021). Achieving the critical view of safety in the difficult laparoscopic cholecystectomy: a prospective study of predictors of failure. Surg Endosc.

[12] Sanjay P, Fulke JL, Exon DJ (2010). 'Critical view of safety' as an alternative to routine intraoperative cholangiography during laparoscopic cholecystectomy for acute biliary pathology. J Gastrointest Surg.

[13] Khan KS, Sajid MA, McMahon RK, Mahmud S, Nassar AH (2020). Hartmann's pouch stones and laparoscopic cholecystectomy: the challenges and the solutions. JSLS.

[14] Jalil T, Adibi A, Mahmoudieh M, Keleidari B (2020). Could preoperative sonographic criteria predict the difficulty of laparoscopic cholecystectomy?. J Res Med Sci.

[15] Bourgouin S, Mancini J, Monchal T, Calvary R, Bordes J, Balandraud P (2016). How to predict difficult laparoscopic cholecystectomy? Proposal for a simple preoperative scoring system. Am J Surg.

[16] Shimoda M, Udo R, Imasato R, Oshiro Y, Suzuki S (2021). What are the risk factors of conversion from total cholecystectomy to bailout surgery?. Surg Endosc.

[17] Vettoretto N, Saronni C, Harbi A, Balestra L, Taglietti L, Giovanetti M (2011). Critical view of safety during laparoscopic cholecystectomy. JSLS.

[18] Mahmud S, Masaud M, Canna K, Nassar AH (2002). Fundus-first laparoscopic cholecystectomy. Surg Endosc.

[19] Bairoliya K, Rajan R, Sindhu RS, Natesh B, Mathew J, Raviram S (2020). Is a difficult gallbladder worth removing in its entirety? - outcomes of subtotal cholecystectomy. J Minim Access Surg.

[20] Toro A, Teodoro M, Khan M, Schembari E, Di Saverio S, Catena F, Di Carlo I (2021). Subtotal cholecystectomy for difficult acute cholecystitis: how to finalize safely by laparoscopy-a systematic review. World J Emerg Surg.

[21] Elshaer M, Gravante G, Thomas K, Sorge R, Al-Hamali S, Ebdewi H (2015). Subtotal cholecystectomy for "difficult gallbladders": systematic review and meta-analysis. JAMA Surg.

